# In Vitro Characterization of Probiotic Potential of *Limosilactobacillus fermentum* against *Salmonella* Gallinarum Causing Fowl Typhoid

**DOI:** 10.3390/ani13081284

**Published:** 2023-04-08

**Authors:** Adnan Mehmood, Muhammad Nawaz, Masood Rabbani, Muhammad Hassan Mushtaq

**Affiliations:** 1 Institute of Microbiology, University of Veterinary and Animal Sciences, Lahore 54000, Pakistan; 2 Department of Epidemiology and Public Health, University of Veterinary and Animal Sciences, Lahore 54000, Pakistan

**Keywords:** fowl typhoid, *Salmonella* Gallinarum, probiotics, *Limosilactobacillus fermentum*

## Abstract

**Simple Summary:**

Fowl typhoid is an infectious bacterial disease of poultry that causes significant economic losses in the poultry sector. Antimicrobial therapy, biosecurity practices and vaccination programs are used to prevent and treat fowl typhoid. Misuse of antibiotics generally results in emergence of multidrug resistance in *Salmonella* Gallinarum. Although vaccination reduces flock losses, disease is still prevailing in many developing countries including Pakistan. To develop an alternative to these approaches, the present study was designed to explore *Lactobacillus* spp. of poultry origin which can inhibit *Salmonella* Gallinarum in vitro. Twenty one lactobacilli isolated from poultry have antibacterial activity against *Salmonella* Gallinarum. These isolates were further selected for the characterization of in vitro probiotic properties. Out of 21, two *Limosilactobacillus fermentum* strains isolated in this study have the ability to survive physicochemical barriers of the gut such as tolerance to low pH, bile salts, auto-aggregation and co-aggregation activity with *Salmonella* Gallinarum, and caused significant reduction in growth of *Salmonella* Gallinarum. These isolates may be used for the development of anti-*Salmonella* Gallinarum probiotics after in vivo study in broilers.

**Abstract:**

Fowl typhoid, a septicaemic disease of poultry, is caused by *Salmonella* Gallinarum and leads to severe economic losses. The aim of the present study was to isolate, select and characterize indigenous probiotic lactobacilli with anti-*Salmonella* Gallinarum activity. A total 55 lactobacilli were isolated from the caeca and ileum parts of healthy chickens and identified to species level by 16S rDNA sequencing. All the isolates were initially screened for antimicrobial activity and selected isolates were further subjected to in vitro evaluation of probiotic properties. Lactobacilli isolates (*n* = 21) showed varying levels of activity (08–18 mm) against *Salmonella* Gallinarum. These selected isolates also showed tolerance to acidic conditions (pH 3 and 4). Out of these 21 isolates, 13 showed growth (>0.5 OD at 600 nm) 0.3% bile salts. Moreover, these isolates also had the ability of auto-aggregation (20.05 ± 0.62%–50.70 ± 1.40%), and co-aggregation with *Salmonella* Gallinarum (5.22 ± 0.21%–42.07 ± 0.70%). Results revealed that lactobacilli had a higher level of resistance to vancomycin (100%), streptomycin (100%), ciprofloxacin (95%), gentamicin (90%), doxycycline (90%), oxytetracycline (85%), and bacitracin (80%), and a lower level of resistance to penicillin (33%), erythromycin (28%), chloramphenicol (23%), fusidic acid (23%) and amoxicillin (4%). The *Limosilactobacillus fermentum* PC-10 and PC-76 were sensitive to most of the antibiotics. The overall results revealed that two *Limosilactobacillus fermentum* strains (PC-10 and PC-76) fulfill the in vitro selection criteria of probiotics, i.e, tolerance to low pH, resistance to bile salts, auto-aggregation, co-aggregation with *Salmonella* Gallinarum, and absence of acquired antibiotic resistance. The *Limosilactobacillus fermentum* PC-10 and PC-76 also inhibited the (>5 log10) growth of *Salmonella* Gallinarum in co-culture assay. It is concluded that *Limosilactobacillus fermentum* PC-10 and PC-76 may be further investigated and developed as anti-*Salmonella* Gallinarum probiotics for poultry.

## 1. Introduction

The poultry industry is one of the most dynamic sectors of livestock in Pakistan. It is developing at an impressive growth rate of 7.5 percent during the last few years and provides employment to about 1.5 million people. Poultry meat contributes more than 40% of the total meat production in Pakistan [1]. The poultry sector is still facing the problem of fowl typhoid, which is a severe septicaemic infection of poultry caused by *Salmonella enterica* serovar Gallinarum. However, this disease has been eradicated from commercial poultry in many developed countries such as Japan, Australia and Canada, and others in Western Europe and North America [2]. In developing countries, including Pakistan, fowl typhoid is endemic and found in both growing and adult chickens [3]. It can cause 10–90% mortality in chickens which eventually leads to massive economic losses in the poultry sector [4].

In the poultry sector, various approaches such as antibiotic therapy, biosecurity plans and vaccines are currently being used for the prevention and control of fowl typhoid [5]. Vaccination is one of the most effective and practical strategies to control this disease [6]. The killed vaccines produce negligible cell-mediated immune response and therefore cannot be used in place of the live vaccines against *Salmonella* Gallinarum. The live vaccine developed from the attenuation of *Salmonella* Gallinarum strain SG9R is widely used for the control of fowl typhoid. The continuous impediment of using live vaccine is the possibility of reversion of the SG9R to pathogenic phenotype due to a point mutation in the *rfaJ* gene. Moreover, the intact plasmid of *Salmonella* Gallinarum SG9R also harbors various virulence genes that can cause fowl typhoid in chicks at a young stage or in immunocompromised brown-egg-laying hens [7].

The extensive misuse of antibiotics in chicken has led to the development and transmission of antibiotic resistance in bacteria such as extended spectrum beta lactams (ESBL), which is one of the major problems for both poultry industry and public health [5]. Emergence of antibiotic-resistant strains and restrictions on antibiotic use in poultry have compelled scientists to find alternate approaches such as probiotics in order to increase production and also to improve disease resistance [8].

According to the Food and Agriculture Organization (FAO), probiotics are defined as ‘live microorganisms which when given in appropriate amounts provide health advantage to the host [9]. *Lactobacillus* is one of the genera most commonly used as probiotics to improve intestinal health and production in poultry [10]. Lactobacilli are common inhabitants of the gut of animals and have acquired Generally Recognized as Safe (GRAS) status. Lactobacilli can inhibit the growth of enteric pathogens by producing organic acids, hydrogen peroxide and bacteriocins. Production of organic acids lowers the pH in the microenvironment of the gut and makes it unsuitable for most pathogenic bacteria, such as *Salmonella*. The bacteriostatic proteins, such as bacteriocins, synthesized by lactobacilli also inhibit the growth of many pathogens. In addition, most of these bacteriocins are pH-stable and are not affected by fluctuation in acidity in different parts of the gut [11]. Since the probiotic properties of lactobacilli are strain-specific and novel environments may have novel probiotic strains, the current study was designed as the first step of a multistep project to isolate and characterize the lactobacilli from caeca and ileum of indigenous poultry. The isolates were screened for their antibacterial effect against *Salmonella* Gallinarum and other desirable probiotic traits, i.e., tolerance to low pH and bile salts activity, absence of acquired antibiotic resistance, auto-aggregation, co-aggregation, and inhibition of *Salmonella* Gallinarum in broth culture in an effort to develop indigenous probiotics for poultry.

## 2. Materials and Methods

### 2.1. Sample Collection

Samples including liver, spleen and caeca were collected from backyard (Golden Misri) and commercial poultry (broiler) in different areas of Punjab Province, i.e., Lahore, Arifwala, Pakpattan and Okara where outbreak of fowl typhoid was reported. Postmortem examination of dead and sick birds suspected of fowl typhoid was conducted and infected liver, spleen and caeca organs were collected aseptically in sterile containers. Samples were kept in an icebox to maintain the cold chain during transportation to the Institute of Microbiology, University of Veterinary and Animal Sciences (UVAS), Lahore.

### 2.2. Isolation of Salmonella Gallinarum

The samples (1 gm) were added to freshly prepared 5 mL selenite broth tubes for the enrichment of *Salmonella* followed by incubation under aerobic conditions at 37 °C for 24 h. The liver and spleen tissues were homogenized before enrichment. After the enrichment, contents (1 mL) from the selenite broth tube were cultured on Salmonella Shigella (SS) agar. After incubation of SS agar plates, presumptive black-colored bacterial colonies were selected from SS agar plates and were subcultured for purification [12]. The isolates were identified by microscopic (Gram staining) and biochemical characterization (motility test, indole production test, Voges–Proskauer test, catalase test, H_2_S production test, methyl red test, oxidase test, citrate utilization test and urease test) following the Bergey’s Manual of Determinative Bacteriology [13,14].

### 2.3. Identification of Salmonella Genus

The Salmonellae genus was confirmed by polymerase chain reaction (PCR) using a 30-bp forward primer (F-5′CGGAACGTTATTTGCGCCATGCTGAGGTAG3′) and a 27-bp reverse primer (R-5′GCATGGATCCCCGCCGGCGAGATTGTG3′) targeting the *hilA* gene [15]. The *Salmonella* Gallinarum was confirmed by amplifying the *glgC* gene using the serovar specific primers (F-5′GATCTGCTGCCAGCTCAA-3′) and (R-5′GCGCCCTTTTCAAAACATA3′) as described previously [16]. The 25 µL PCR reaction mixtures contained nuclease-free water (7.5 µL), 12.5 µL of DreamTaq Green PCR master mix 2× (Thermo Scientific, Waltham, MA, USA) and 1.5 µL of each of the forward and reverse primers (10 µM) and template DNA (2 µL). The amplification was performed by the following program: 1 cycle of 94 °C for 5 min, followed by 30 cycles of denaturation at 94 °C for 30 s, annealing at 55 °C for 30 s and extension at 72 °C for 30 s, and a final extension at 72 °C for 7 min [16].

### 2.4. Isolation and Preliminary Identification of Lactobacilli

Samples of caeca (*n* = 30) and ileum (*n* = 30) of healthy backyard (Golden Misri) poultry from flocks having an outbreak of fowl typhoid were collected in a sterile container from different areas of Punjab. The contents of caeca and ileum were transferred aseptically into normal saline tubes and 10-fold serial dilutions were made. The lactobacilli were cultured on De Man Rogosa Sharpe (MRS) media plates supplemented with fluconazole (100 µg/mL). Plates were incubated under anaerobic conditions at 37 °C for 48 h. Colonies with different morphological characteristics were selected from the culture plates for Gram staining and catalase test. After confirmation, selected colonies were purified by subculturing two or three times on MRS agar and then stored in MRS broth (Hi Media) with 30% glycerol added [17]. 

### 2.5. Identification of Lactobacilli by Molecular Method

The isolates identified as Gram-positive rods and catalase negative were confirmed by *Lactobacillus* genus-specific PCR. The DNAs of all the isolates were extracted by a commercial DNA extraction kit (Fair Biotech) following the instructions of the manufacturer (Fair Biotech, Taiwan, China). The isolates were identified to genus level by genus-specific PCR using forward primer XB5 (5′GCCTTGTACACACCGCCCGT′3) and reverse primer LBLMA-I (5′CTCAAAACTAAACAAAGTTC′3), and to species level by amplifying the 16S rRNA gene using universal primers (8FLP and XB4) as described previously [18]. Briefly, the reaction mixture (25 µL) contained 12.5 µL of 2× master mix, 1.5 µL of 10 µM of respective primers, 7.5 µL of nuclease-free water and template DNA (2 µL). The amplification was done using Simpliamp Thermal Cycler (Applied Biosystems, Waltham, MA, USA) using the following program: initial denaturation at 94 °C for 10 min followed by 35 cycles of denaturation at 94 °C for 1 min, annealing at 55 °C for 1 min, elongation at 72 °C for 1 min and a final elongation step at 72 °C for 10 min. The amplicons of genus-specific amplification (250 bp) and 16S rRNA gene (1400 bp) were resolved on 1.5% agarose gel electrophoresis and sequenced by a commercial sequencing service (Macrogen, Seoul, South Korea). The sequences were analyzed by BioEdit and species were identified by comparing the retrieved sequences with the NCBI GenBank database using the Basic Local Alignment Search Tool [19]. The sequences were also submitted to NCBI GenBank and their accession numbers were obtained.

### 2.6. Antagonistic Effect of Lactobacilli Isolates on Salmonella Gallinarum

Antibacterial activity of lactobacilli was determined by well diffusion assay as described previously. Briefly, freshly grown cultures of lactobacilli in MRS broth were centrifuged at 10,000 rpm for 5 min and supernatant was collected. Cell-free supernatants (CFSs) were prepared by filtration of supernatants through a 0.22 µm syringe filter. The CFSs were divided into two parts and the pH of one part was adjusted as 7.0. The standard inoculum (0.5 McFarland) of *Salmonella* Gallinarum prepared in normal saline was swabbed on Mueller Hinton Agar plates with a sterile swab. The wells were made and sealed with molten agar. The CFSs (100 µL) were added in wells and plates were incubated at 37 °C for 24 h under aerobic conditions. Antibacterial activity was determined as the diameter of the zone of inhibition (mm) around the wells [20].

### 2.7. In Vitro Determination of Probiotic Properties of Lactobacilli

#### 2.7.1. Tolerance to Low pH

The tolerance of lactobacilli to low pH was determined as described previously [21]. Briefly, exponentially grown isolates were suspended in phosphate buffer saline to prepare bacterial inoculum (containing approximately 3 × 10^8^ CFU/mL). The 100 µL of these suspensions were added to 10mL MRS broth tubes at different pH (pH 3, 4, 7) and incubated for 90 min. After incubation, 10-fold serial dilutions were plated on MRS media plates for enumeration of lactobacilli. Tolerance to low pH was determined by comparing the number of viable cells (CFU/mL) recovered under normal conditions (pH 7) and after exposure to acidic conditions (pH 3 and 4). A mean log10 reduction of more than 0.4 (60%) at pH 3 was considered as a cut-off for tolerance to low pH.

#### 2.7.2. Resistance to Bile Salts

The tolerance of lactobacilli to bile salts was determined as described previously by Bao et al. [20]. Briefly, 200 µL of MRS broth supplemented with different concentrations of bile salts (0.3%, 1% and 1.8%) was added on a 96-well microtiter plate. The inoculum of lactobacilli (equivalent to 1.0 MF) was prepared in MRS broth and 20 µL of the inoculum was added into respective wells of the microtiter plate. Plates were incubated at 37 °C for 24 h and growth of lactobacilli was monitored by measuring the Optical Density (O.D) at 600 nm by a spectrophotometer (Multiskan Sky, Spectrophotometer, Thermoscientific, Waltham, MA, USA) at 0 h and 24 h. An increase in 0.5 OD of the isolates after 24 h growth was considered as good tolerance and survival ability.

#### 2.7.3. Antibiotic Resistance Profile

Antibiotic susceptibility pattern of lactobacilli to different antibiotics was determined by broth microdilution method on a 96-well microtiter plate using susceptibility test media as described previously [22]. Briefly, 100 µL of LSM broth containing doubly diluted antibiotics was added to the 96-well plates. Plates were inoculated with 100 µL (10^5^ CFU/mL) of inoculum of respective lactobacilli. Inoculum of lactobacilli was prepared by suspending the exponentially grown isolates from MRS plates into normal saline and adjusting the OD equivalent 1 McFarland and then further diluting (1:1000) it into LSM broth. Plates were incubated at 37 °C for 24 h. Minimum inhibitory concentrations (MICs) of the antibiotics were read as the lowest concentration of antibiotic which resulted in no visible growth. On the basis of MICs, the isolates were designated as resistant, intermediate or sensitive following the breakpoints and guidelines provided by the European Food Safety Authority or as described previously [18,23].

#### 2.7.4. Auto-Aggregation and Co-Aggregation Assay

Auto-aggregation represents the ability of the strain to clump together and indirectly reveal its adhesion capacity to gut epithelium while co-aggregation indicates the ability to clump with other bacterial strains and exert its inhibitory effect. Auto-aggregation and co-aggregation activity of lactobacilli were analyzed by following the method as described previously [20]. Briefly, for the determination of auto-aggregation activity, freshly grown lactobacilli isolates in MRS broth were centrifuged at 6000 rpm for 15 min. The supernatants were discarded and pellets were washed and resuspended in phosphate buffer saline (OD equal to 1 McFarland) followed by incubation at 37 °C up to two hours. After incubation, optical densities were measured at 600 nm and percentage auto-aggregation capacities of the isolates were calculated by following the formula:

%auto-aggregation = A_0_ − A_t_/A_0_ × 100

where A_0_ represents the OD value of an isolate at 0 h and A_t_ is the OD value after incubation for a specific time.

To determine the co-aggregation of lactobacilli with *Salmonella* Gallinarum, equal volumes (1 mL) of lactobacilli and *Salmonella* Gallinarum suspensions in PBS (equal to I McFarland) were mixed and incubated at 37 °C. Similarly, lactobacilli and *Salmonella* Gallinarum were also resuspended in PBS separately and incubated in the same conditions. Optical densities were measured at 600 nm at different time intervals (1 h and 2 h) and co-aggregation was determined by using the following formula:

%Co-aggregation = (OD_1_ + OD_2_) − 2(OD3)/(OD_1_ + OD_2_) × 100

where OD_1_ represents the value of strain 1 (lactobacilli), OD_2_ is the value of strain 2 (*Salmonella* Gallinarum) and OD_3_ is the value of mixture of strain 1 and 2. The auto-aggregation and co-aggregation of the isolates were analyzed using *Lactobacillus rhamnosus* GG (LGG), procured from the Institute of Microbiology, UVAS, Lahore, as control

#### 2.7.5. Inhibition of *Salmonella* Gallinarum in Broth Culture

To study the growth kinetics of *Salmonella* Gallinarum and selected lactobacilli in co-culture conditions, 1 mL of each of freshly grown culture of *Salmonella* Gallinarum and selected *Lactobacillus* isolate were added to 10 mL of nutrient broth and incubated at 37 °C for 24 h. Both of the *Lactobacillus* isolates and *Salmonella* Gallinarum were enumerated at different time intervals (0, 6, 12 and 24 h) on MRS agar and salmonella shigella agar, respectively [24]. The counts were converted into mean log_10_ CFU/mL and log10 reduction of *Salmonella* Gallinarum was determined.

### 2.8. Statistical Analysis

The data of optical density and enumeration were presented as Mean ± Standard Deviation (SD) and statistically significant differences among isolates were determined by one-way ANOVA followed by post hoc Tukey’s Multiple Comparison Test using graph pad prism 8.0 software.

## 3. Results

### 3.1. Isolation and Identification of Salmonella Gallinarum

In the present study, *Salmonella* Gallinarum (*n* = 5) were isolated from field outbreaks in different districts of Punjab. The black centered colonies, typical of *Salmonella*, were observed on SS agar after culturing the samples. The selected Salmonellae (*n* = 50) were small Gram-negative rods, in single or in pair form. Biochemical tests revealed that all the salmonellae were positive for catalase, oxidase, methyl red, citrate utilization and triple sugar iron test while negative to indole, urease and VP test. All isolates were confirmed by genus-specific PCR of *Salmonella*. Out of 50 salmonellae, five isolates were identified as non-motile and assumed to either belong to *Salmonella* Pullorum or *Salmonella* Gallinarum. All these five non-motile *Salmonella* were identified as *Salmonella* Gallinarum by serovar-specific PCR which amplified a 194 bp region of *glgC* gene.

### 3.2. Isolation and Identification of Lactobacilli Isolates

**Figure 1:**
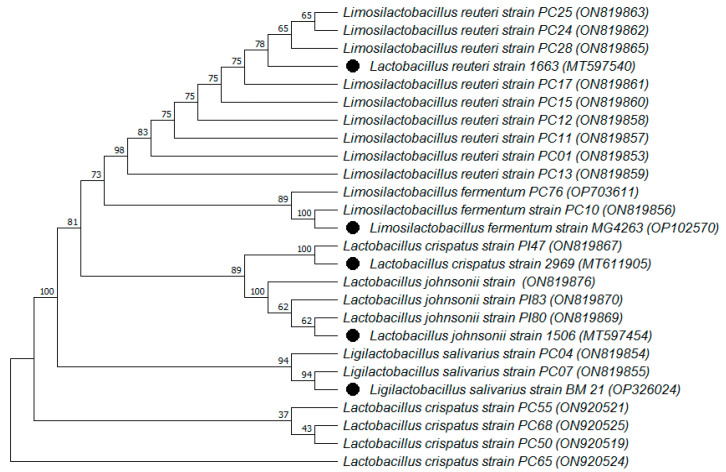
A phylogenetic tree was constructed by using the neighbor joining method. It shows the genetic resemblance of *Lactobacillus* species isolated in this study with reference strains based on their partial 16S rRNA gene sequence. Reference strains represented with filled circles were taken from the NCBI database. Branches indicate the bootstrap percentage after 1000 replications. Codes in parenthesis are GenBank accession numbers of the isolates.

Poultry caeca and ileum samples were cultured on MRS agar plates and a total of 95 isolates (catalase-negative, Gram-positive rods) were selected as presumptive lactobacilli. Out of these 95, 55 isolates were confirmed as lactobacilli by genus-specific PCR. Out of 55, 21 selected isolates were further identified to species level by sequencing their partial 16S rRNA gene or 16S rDNA-23SrDNA interspacer region. The sequencing results revealed that the selected lactobacilli were *Limosilactobacillus reuteri* (09), *Ligilactobacillus salivarius* (02), *Limosilactobacillus fermentum* (02), *Lactobacillus crispatus* (05) and *Lactobacillus johnsonii* (03). The GenBank accession numbers of these lactobacilli are ON819853-ON819863, ON819865, ON819867, ON819869, ON819870, ON819876, OP703611, ON920521, ON920522, ON920524 and ON920525. An evolutionary tree constructed by the neighbor joining method on the basis of 16SrRNA gene sequences of the selected isolates is given in Figure 1.

### 3.3. Antibacterial Activity of Cell Free Supernatants of Lactobacilli

**Figure 2:**
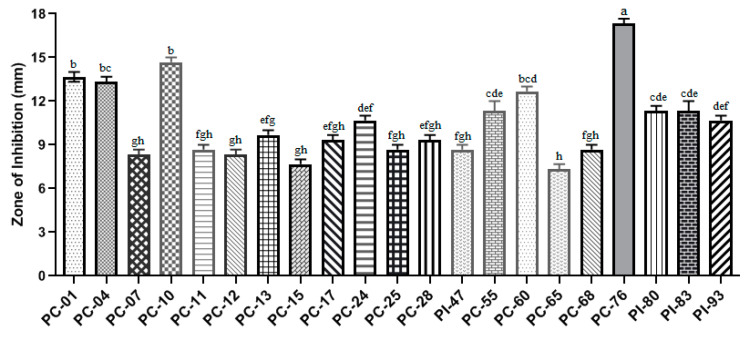
Anti-*Salmonella* Gallinarum activity of cell free supernatants of lactobacilli. ^a–h^ Bars represented with the same alphabet shows the non-significant difference (*p* > 0.05) between isolates while bars with different alphabets show the significant difference (*p* < 0.05) between isolates.

Activity of the CFSs of all lactobacilli isolates directly and after adjusting the pH (7.0) was determined against *Salmonella* Gallinarum by well diffusion assay. Out of 55, CFSs of 21 lactobacilli isolates when used directly showed activity against *Salmonella* Gallinarum. The CFSs of PC-76 and PC-10 showed higher antimicrobial activity (18 ± 0.5 mm and 15 ± 0.5 mm, respectively) as compared to CFSs of other isolates. Activity of CFSs of all isolates against *Salmonella* Gallinarum is shown in Figure 2.

### 3.4. Tolerance to Acidic pH

The effect of acidic conditions (pH 4, 3) on the viability of selected 21 lactobacilli isolates is shown in Table 1. All isolates showed more microbial growth at pH 7 and exhibited varying levels of tolerance to acidic pH (3 and 4). The isolates PC-07 and PC-17 showed significantly higher growth rate (*p* < 0.05) at pH 4 (mean log10 CFU/mL 7.83 ± 0.1 and 7.78 ± 0.1, respectively) compared to other isolates, while PI-47 exhibited less growth (mean log10 CFU/mL 5.49 ± 0.1). Moreover, at pH 3, PC-07 and PC-13 showed higher growth rate (mean log10 CFU/mL 7.84 ± 0.1, 7.83 ± 0.1) while PC-65 and PI-47 exhibited lower tolerance to acidic pH, as is evidenced by a decrease in mean log10 CFU/mL 4.69 ± 0.1 and 4.84 ± 0.1, respectively. Out of 21, 14 isolates (PC-01, PC-04, PC-11, PC-15, PC-17, PC-28, PC-47, PC-55, PC-60, PC-65, PC-68, PI-80 and PI-83, PI-93) showed poor tolerance to acidity (pH 3) as marked by more than 0.4 log reduction in their counts as compared to pH 7. The isolates PC-07, PC-10, PC-12, PC-13, PC-24, PC-25 and PC-76 showed good tolerance to pH 3 (<0.4 log reduction). Remarkably, few isolates (PC-12, PC-13, PC-15, PC-76 and PI-83) showed better tolerance to pH 3 as compared to pH 4.

### 3.5. Bile Salt Tolerance of Lactobacilli

**Figure 3:**
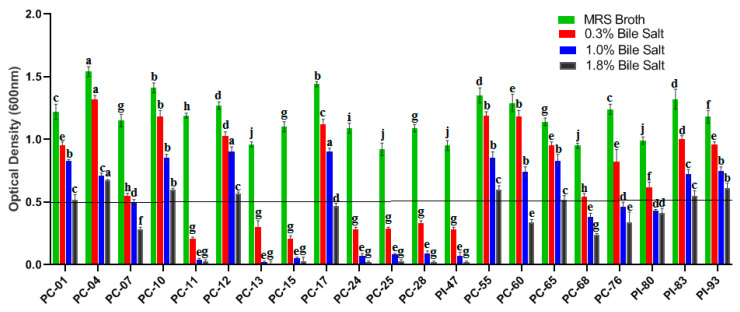
Growth of lactobacilli in MRS broth containing different concentrations of bile salts. Bars represented with the same alphabet shows a non-significant difference (*p* value > 0.05) between isolates while bars represented with a different alphabet shows significant difference (*p* value < 0.05) between isolates at different concentrations of bile salt.

The lactobacilli isolates showed variable levels of tolerance and growth in MRS broth supplemented with different concentrations of bile salts, as shown in Figure 3. All of the isolates showed higher growth in MRS broth, followed by MRS broth supplemented with 0.3%, 1.0% and 1.8% bile salts. The isolate PC-04 showed higher growth (OD 1.32 ± 0.03) at 0.3% bile salt concentration followed by PC-10, PC-55, PC-60 (OD 1.18 ± 0.05, 1.19 ± 0.03, 1.18 ± 0.05), respectively. The PC-12 and PC-17 showed highest tolerance (OD 0.92 ± 0.03, 0.90 ± 0.03) when exposed to 1% bile salt concentration. At a higher concentration of bile salts (1.8%), lactobacilli isolate PC-04 exhibited the higher tolerance (OD 0.67 ± 0.01) and was meaningfully different (*p* < 0.05) among all other lactobacilli isolates. Out of 21 isolates, only 13 (PC-01, PC-04, PC-07, PC-10, PC-12, PC-17, PC-55, PC-60, PC-65, PC-76, PI-80, PI-83 and PI-93) isolates showed tolerance and growth (>0.5 OD) at 0.3% bile salts, while PC-01, PC-04, PC-10, PC-12, PC-55, PC-65, PI-83 and PI-93 also showed good growth in MRS broth supplemented with 1.8%.

### 3.6. Auto-Aggregation and Co-Aggregation Activity of Lactobacilli

Auto-aggregation and co-aggregation activity of selected lactobacilli isolates is given in Table 2. The auto-aggregation activity of isolates after 2 h was in the range of 20.05 ± 0.62% to 50.70 ± 1.40%. The highest auto-aggregation (*p* < 0.05) was observed for PC-10 (50.70 ± 1.40%) followed by PC-11 (46.75 ± 3.12%), while the lowest auto-aggregation activity was noted for PC-01 (20.05 ± 0.62%) in comparison to control LGG isolate (39.00 ± 0.20%). All the isolates exhibited variable co-aggregation activity with *Salmonella* Gallinarum after 2 h (5.22 ± 0.21–42.07 ± 0.70%). The PC-76 showed significantly higher (*p* < 0.05) co-aggregation activity after 1 h (29.15 ± 0.72%) and 2 h (38.30 ± 0.45%) compared to other isolates and significantly lower with LGG isolate (32.30 ± 0.55, 42.07 ± 0.70%, respectively).

### 3.7. Antibiotic Susceptibility Profile

The MICs of different antibiotics were determined by the broth microdilution method and results were interpreted using the breakpoints recommended by the European Food Safety Authority. The results of the study revealed that lactobacilli had higher levels of resistance to vancomycin (21/21, 100%), streptomycin (21/21, 100%), ciprofloxacin (20/21, 95%), gentamicin (19/21, 90%), doxycycline (19/21, 90%), oxytetracycline (18/21, 85%) and bacitracin (17/21, 80%), and lower levels of resistance to penicillin (7/21, 33%), erythromycin (6/21, 28%), (16/21, 76%), chloramphenicol (05/21, 23%), fusidic acid (05/21, 23%) and amoxicillin (01/21, 04%). Resistance phenotypes of each isolate are given in Table 3. *Limosilactobacillus fermentum* PC-10 and PC-76 were sensitive to most of the antibiotics. The MICs value of different antibiotics against lactobacilli are given in Supplementary data file Table S1.

### 3.8. Limosilactobacillus fermentum PC-10 and PC-76 Inhibit the Growth of Salmonella Gallinarum in Co-Culture Assays

**Figure 4:**
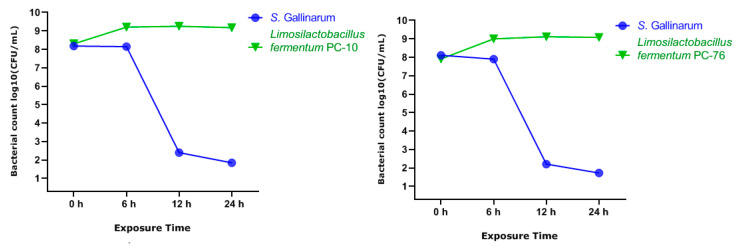
Enumeration of *Salmonella* Gallinarum and *Limosilactobacillus fermentum* PC-10 and PC-76 at specific times in co-culture assay.

*Limosilactobacillus fermentum* PC-10 and PC-76 were co-cultured with *Salmonella* Gallinarum in nutrient broth and their counts were determined at 0, 6, 12 and 24 h using respective selective media. The kinetics in terms of mean log10 CFU/mL of the PC-10, PC-76 and *Salmonella* Gallinarum are presented in Figure 4. Results revealed that PC-10 caused a non-significant (*p* > 0.05) decrease in *Salmonella* Gallinarum count after 6 h (mean log10 CFU/mL 8.14 ± 0.10) and a remarkable reduction (*p* < 0.05) after 12 and 24 h (mean log10 CFU/mL 2.39 ± 0.05 and 1.84 ± 0.01, respectively) as compared to initial counts (mean log10 CFU/mL 8.17 ± 0.15). Similarly, PC-76 also reduced the viable counts of *Salmonella* Gallinarum at 6, 12 and 24 h (mean log10 CFU/mL 7.90 ± 0.25, 2.20 ± 0.18, and 1.72 ± 0.10, respectively) as compared to its initial counts (mean log10 CFU/mL 8.11 ± 0.31). Following longer incubation, the *Limosilactobacillus fermentum* PC-10 and PC-76 reduced the pH of media to 3.7 and 3.9, respectively, causing a remarkable reduction (>6 log) in *Salmonella* Gallinarum counts in 24 h.

## 4. Discussion

Antibiotics are commonly used for the control and treatment of fowl typhoid and other bacterial infections of poultry. The overuse and misuse of antibiotics has resulted in the emergence of antibiotic resistance in bacteria. Transmission of antibiotic-resistant bacteria from poultry to the human food chain is one of the major risks to public safety [25]. Therefore, it is urgent to explore alternative strategies such as probiotics for the control of bacterial infections of poultry. Bacterial species belonging to the genus *Lactobacillus* are the most commonly used poultry probiotics. Lactobacilli do not pose any harmful effect on the host and are Generally Recognized as Safe (GRAS). *Lactobacillus* spp. are natural inhabitants of green plants, fermented foods and the gastrointestinal tract of humans and animals, and can be employed in the food industry for medical and therapeutic purposes [26,27]. The naturally resident microbiota of chicken gut is comprised of a large diversity of microbes, and is an excellent source for the selection of effective probiotic strains [28].

In this study, a total of 55 lactobacilli were isolated from the caeca and ileum of healthy chicken in flocks having an outbreak of fowl typhoid. The rationale behind selecting such flocks was that if a chicken remains healthy during the outbreak of fowl typhoid, it might have superior microbiota which might be explored for the selection of probiotics. Out of 55, 21 lactobacilli were selected for further analysis on the basis of their ability to inhibit *Salmonella* Gallinarum. Various research studies have isolated lactic acid bacteria from fermented foods, intestinal contents and droppings of poultry and analyzed their antimicrobial activity against *Salmonella enteritidis* [29,30,31]. The species-level identification of 21 lactobacilli selected in this study revealed that these were *Limosilactobacillus reuteri*, *Ligilactobacillus salivarius*, *Limosilactobacillus fermentum*, *Lactobacillus crispatus* and *Lactobacillus johnsonii*. Our findings are similar to previous studies which have also reported that lactobacilli including *L. reuteri*, *L. johnsonii*, *L. crispatus*, *L. acidophilus*, *L. salivarius* and *L. aviaries* are most common inhabitants of the poultry gut [32,33]. Inhibitory effect of probiotics against gut pathogens is considered another important characteristic in the selection of effective probiotic strains [34]. Since the neutralized CFSs of the lactobacilli reported in this study showed no inhibitory activity against the *Salmonella* Gallinarum, it is assumed that the activity of these isolates was because of the production of organic acids. It has also been reported previously that acid neutralization of CFSs of lactobacilli can result in loss of their antibacterial activity [29]. Other than lactobacilli, *Bacillus subtilis* strains have also been reported to have activity against *Salmonella* Gallinarum [35].

Although the in vitro characterization of potentially beneficial microbes cannot entirely mimic gastrointestinal conditions, it still is considered a cheap, reliable and fast method for screening of large microbial populations for desired beneficial properties [34]. One of the essential probiotic properties of lactic acid bacteria is their viability at the acidic pH of the stomach and ability to withstand the high concentration of bile salts. These characteristics are regarded as positive indicators for the survival of bacteria in the gut [36]. This study revealed a significant loss in viability of most of the strains (14/21) after 90 min exposure to an acidic environment (pH 3 and pH 4). Only seven (33.33%) strains which showed <0.4 log or <60% reduction in viability were considered acid-tolerant strains. Similar to the findings of this study, a previous report also recovered more than 50% of lactobacilli at pH 3 [37]. It has also been reported previously that lactobacilli are less tolerant to pH 3 and pH 2 as compared to pH 4, which is in accordance with the results of the current study [30]. The concentration of bile salt in the poultry gut is in the range of 0.1–1.0% and following the standard criteria, probiotic strains should be able to survive at 0.10 to 0.30% bile salt concentrations [38,39]. Results similar to the findings of this study have also been reported previously [17,30,40]. Since the probiotics should tolerate and grow in good numbers in the gut to exert their beneficial effect, an increase of at least 0.5 in optical density at 0.3% bile salt concentration was considered a cut-off as selection criteria. Most of the isolates (13/21) were able to grow well in 0.3% bile salts, while 11 were resistant to 1.0% bile salts and eight were resistant to 1.8% bile salts.

Auto-aggregation is another property of lactobacilli which depicts its ability to form a biofilm that protects the host from invading pathogens. This activity also indirectly reveals the capacity of lactobacilli to attach to the surface of intestinal epithelial cells [41]. Co-aggregation of lactobacilli with pathogens indicates their ability to attach with pathogens in vivo and create a microenvironment where their antimicrobial metabolites can inhibit the pathogens [42]. In this study, auto-aggregation activity and co-aggregation of lactobacilli with *Salmonella* Gallinarum were in the range of 20.05 ± 0.62%–50.70 ± 1.40% and 5.22 ± 0.21%–42.07 ± 0.70%, respectively. Another study recorded higher co-aggregation of lactic acid bacteria with pathogenic *Staphylococcus aureus* (10.15–38.5%) while their results showed the low co-aggregation (5.56–8.62%) activity of *L. fermentum* with *E. coli* 0157H7 [43].

Various studies indicated that antibiotic resistance genes of lactobacilli can be passed to resident organisms in host GIT. So, considering the safety issue of probiotics, antibiotic resistance patterns of isolates were investigated. The *Lactobacillus* species are normally resistant to antibiotics that inhibit DNA synthesis (quinolones). They are sensitive to all cell wall and protein synthesis inhibitors except vancomycin and aminoglycoside [44]. A higher level of resistance to aminoglycoside and vancomycin, ciprofloxacin and tetracycline is reported in lactobacilli isolates in this study which is in accordance with the previous studies. Lactobacilli resistant to erythromycin, penicillin or tetracycline are considered a threat to public safety as the resistance to these antibiotics is generally acquired in lactobacilli. The isolates showing the acquired resistance were rejected for probiotic potential. The *Limosilactobacillus fermentum* PC-10 and PC-76 were sensitive to most of the antibiotics and did not contain the acquired resistance; therefore, these are safe for addition to the food chain and pose no risk of antibiotic resistance transfer. Many previous studies have also reported similar results of antibiotic resistance in lactobacilli from poultry or food chain [8,29].

The co-culture assay is used to assess the antagonistic effect of one organism on the growth of another organism, when both are cultured together in broth [45]. In this study, *Limosilactobacillus fermentum* PC-10 and PC-76 were selected for co-culture assay on the basis of their antimicrobial activity against *Salmonella* Gallinarum in well diffusion assay, their probiotic potential, and absence of resistance to most of the antibiotic studies. The results revealed that both of the lactobacilli strains can significantly inhibit (>5 log reduction) the growth of *Salmonella* Gallinarum. Similar results have also been reported previously where strains of *L. fermentum* and *L. gasseri* inhibited the growth of *Salmonella* Enteritidis [29] Therefore, the isolated strains of lactobacilli in the present study, *Limosilactobacillus fermentum* PC-10 and PC-76, can be used to develop a probiotic product after in vivo studies in chicken. To the best of our knowledge, this is the first study in Pakistan which analyses the in vitro probiotic properties of *Limosilactobacillus fermentum* against *Salmonella* Gallinarum. Two novel strains (PC-10 and PC-76) isolated from the natural habitat of chicken have the ability to inhibit the growth of *Salmonella* Gallinarum and have suitable in vitro probiotic properties. These isolates can be employed in poultry after studying their beneficial effect in broilers.

## 5. Conclusions

It is concluded that chicken-derived *Limosilactobacillus fermentum* (PC-10 and PC-76) have in vitro probiotic potential and antagonistic activity against *Salmonella* Gallinarum. These strains may be used to develop indigenous probiotics against *Salmonella* Gallinarum infection after in vivo evaluation in chicken.

## Figures and Tables

**Table 1:** Lactobacilli count on MRS agar after treatment in MRS broth of different pH.

Lactobacilli	Means ± SD of Log_10_ Bacterial Counts			Log (Log pH 7–Log pH 3)
	pH7	pH4	pH3	
PC-01	7.71 ± 0.1 ^ab^	7.04 ± 0.1 ^abcde^	6.72 ± 0.2 ^bcd^	0.99
PC-04	8.59 ± 0.2 ^a^	7.67 ± 0.1 ^ab^	6.68 ± 0.1 ^bcd^	1.91
PC-07	7.84 ± 0.1 ^ab^	7.83 ± 0.1 ^a^	7.84 ± 0.1 ^a^	0
PC-10	7.15 ± 0.2 ^b^	7.70 ± 0.2 ^ab^	6.95 ± 0.1 ^ab^	0.2
PC-11	6.74 ± 0.3 ^b^	5.57 ± 0.1 ^fg^	5.40 ± 0.2 ^ef^	1.34
PC-12	7.70 ± 0.4 ^ab^	6.65 ± 0.3 ^bcdef^	7.32 ± 0.4 ^ab^	0.38
PC-13	7.53 ± 0.4 ^ab^	7.14 ± 0.3 ^abcde^	7.83 ± 0.1 ^a^	−0.3
PC-15	7.73 ± 0.3 ^ab^	6.61 ± 0.1 ^bcdef^	7.28 ± 0.2 ^ab^	0.45
PC-17	7.83 ± 0.3 ^ab^	7.78 ± 0.1 ^a^	6.76 ± 0.3 ^abc^	1.07
PC-24	5.28 ± 0.3 ^c^	6.40 ± 0.2 ^cdefg^	5.74 ± 0.1 ^cdef^	−0.46
PC-25	7.38 ± 0.3 ^ab^	7.34 ± 0.1 ^abc^	7.23 ± 0.3 ^ab^	0.15
PC-28	7.34 ± 0.2 ^ab^	7.38 ± 0.3 ^abc^	6.88 ± 0.1 ^ab^	0.46
PI-47	7.00 ± 0.4 ^b^	5.49 ± 0.1 ^g^	4.84 ± 0.1 ^f^	2.16
PC-55	7.9 ± 0.2 ^ab^	7.17 ± 0.3 ^abcd^	7.00 ± 0.1 ^ab^	0.9
PC-60	7.50 ± 0.3 ^ab^	7.11 ± 0.2 ^abcde^	5.65 ± 0.2 ^def^	1.85
PC-65	7.08 ± 0.1 ^b^	5.65 ± 0.1 ^fg^	4.69 ± 0.1 ^f^	2.39
PC-68	7.49 ± 0.1 ^ab^	7.30 ± 0.3 ^abcd^	6.83 ± 0.1 ^ab^	0.66
PC-76	7.30 ± 0.2 ^ab^	6.65 ± 0.1 ^bcdef^	7.00 ± 0.3 ^ab^	0.30
PI-80	7.50 ± 0.3 ^ab^	5.90 ± 0.2 ^fg^	5.58 ± 0.2 ^ef^	1.92
PI-83	7.25 ± 0.2 ^ab^	6.22 ± 0.2 ^defg^	6.26 ± 0.1 ^bcde^	0.99
PI-93	6.70 ± 0.3 ^b^	6.04 ± 0.2 ^efg^	5.41 ± 0.1 ^ef^	1.29

Means that do not share a letter in the same column are significantly different.

**Table 2:** Auto-aggregation and co-aggregation activity of lactobacilli at different time intervals.

Isolates	Percentage Auto-Aggregation Means ± SD		Percentage Co-Aggregation Means ± SD	
	1 h	2 h	1 h	2 h
PC-01	16.22 ± 0.10 ^fg^	20.05 ± 0.62 ^j^	17.20 ± 0.25 ^hi^	19.22 ± 0.35 ^m^
PC-04	22.04 ± 0.31 ^de^	41.23 ± 0.50 ^bcdef^	24.33 ± 0.80 ^c^	35.10 ± 0.72 ^c^
PC-07	25.40 ± 0.16 ^bc^	35.70 ± 0.89 ^fg^	20.44 ± 0.19 ^fg^	29.50 ± 0.52 ^efg^
PC-10	31.10 ± 0.30 ^a^	50.70 ± 1.40 ^a^	24.10 ± 0.42 ^cd^	35.40 ± 1.32 ^bc^
PC-11	22.10 ± 0.29 ^de^	46.75 ± 3.12 ^ab^	21.40 ± 0.25 ^def^	27.01 ± 0.43 ^ghi^
PC-12	13.50 ± 0.45 ^h^	27.60 ± 1.23 ^hi^	20.05 ± 0.73 ^fg^	25.30 ± 0.37 ^ij^
PC-13	30.20 ± 0.45 ^a^	44.90 ± 1.09 ^abc^	08.14 ± 0.15 ^l^	08.50 ± 0.06 ^n^
PC-15	17.30 ± 0.11 ^f^	29.23 ± 0.35 ^hi^	17.19 ± 0.36 ^hi^	23.26 ± 0.85 ^jk^
PC-17	25.32 ± 0.45 ^bc^	42.53 ± 0.22 ^bcd^	23.20 ± 0.81 ^cde^	33.20 ± 0.10 ^cd^
PC-24	14.20 ± 0.21 ^gh^	25.40 ± 1.25 ^ij^	17.24 ± 0.45 ^hi^	22.24 ± 0.29 ^kl^
PC-25	26.15 ± 0.85 ^b^	36.40 ± 0.72 ^efg^	18.28 ± 0.45 ^gh^	28.90 ± 0.10 ^efg^
PC-28	27.20 ± 0.49 ^b^	42.60 ± 0.47 ^bcd^	15.25 ± 0.57 ^ij^	25.45 ± 0.79 ^hij^
PI-47	23.10 ± 0.13 ^cde^	33.40 ± 0.23 ^gh^	16.29 ± 0.65 ^hij^	20.23 ± 0.11 ^lm^
PC-55	25.19 ± 0.66 ^bc^	43.23 ± 1.33 ^bc^	12.39 ± 0.40 ^k^	10.23 ± 0.10 ^n^
PC-60	20.80 ± 0.10 ^e^	36.75 ± 0.80 ^defg^	14.68 ± 0.20 ^ijk^	21.60 ± 0.44 ^klm^
PC-65	14.10 ± 0.13 ^gh^	25.40 ± 0.46 ^ij^	08.39 ± 0.40 ^l^	05.22 ± 0.21 ^o^
PC-68	22.09 ± 0.80 ^de^	42.13 ± 1.33 ^bcde^	16.39 ± 0.34 ^hij^	19.76 ± 0.26 ^lm^
PC-76	25.40 ± 0.32 ^bc^	41.45 ± 1.21 ^bcdef^	29.15 ± 0.72 ^b^	38.30 ± 0.45 ^b^
PI-80	15.10 ± 0.71 ^fgh^	26.70 ± 0.42 ^i^	14.24 ± 0.30 ^jk^	28.40 ± 0.22 ^fgh^
PI-83	21.55 ± 0.27 ^de^	37.10 ± 0.35 ^defg^	21.19 ± 0.21 ^ef^	31.50 ± 0.37 ^de^
PI-93	23.65 ± 0.22 ^cd^	41.70 ± 0.22 ^bcdef^	20.48 ± 0.24 ^efg^	30.42 ± 0.44 ^def^
LGG	15.40 ± 0.30 ^fgh^	39.00 ± 0.20 ^cdefg^	32.30 ± 0.55 ^a^	42.07 ± 0.70 ^a^

Means that do not share a letter in the same column are significantly different.

**Table 3:** Antibiotic resistance profile of selected lactobacilli.

Organisms	Strain	Resistance to Antibiotics
*Limosilactobacillus reuteri*	PC-01	ERY, CIP, S, PEN, OT, VA, CN, BAC, DA
*Ligilactobacillus salivarius*	PC-04	CIP, S, OT, VA, CN, BAC, CHL, DA
*Ligilactobacillus salivarius*	PC-07	CIP, S, PEN, OT, VA, CN, BAC, CHL, DA
*Limosilactobacillus fermentum*	PC-10	CIP, S, VA
*Limosilactobacillus reuteri*	PC-11	CIP, S, OT, VAN, CN, BAC, CHL, DA
*Limosilactobacillus reuteri*	PC-12	ERY, CIP, S, OT, VA, CN, BAC, DA
*Limosilactobacillus reuteri*	PC-13	CIP, S, PEN, OT, VA, CN, BAC, DA
*Limosilactobacillus reuteri*	PC-15	ERY, CIP, S, OT, VA, CN, BAC, DA
*Limosilactobacillus reuteri*	PC-17	ERY, CIP, S, OT, VA, CN, BAC, DA
*Limosilactobacillus reuteri*	PC-24	CIP, S, OT, VA, CN, BAC, CHL, DA
*Limosilactobacillus reuteri*	PC-25	CIP, S, OT, VA, CN, BAC, CHL, DA
*Limosilactobacillus reuteri*	PC-28	CIP, S, PEN, OT, VA, CN, BAC, DA
*Lactobacillus crispatus*	PI-47	CIP, S, PEN, OT, VA, CN, BAC, DA
*Lactobacillus crispatus*	PC-55	CIP, S, PEN, OT, VA, CN, DA
*Lactobacillus crispatus*	PC-60	AML, ERY, CIP, S, CN, FUS, DA
*Lactobacillus crispatus*	PC-65	CIP, S, OT, VA, CN, BAC, FUS, DA
*Lactobacillus crispatus*	PC-68	ERY, CIP, S, OT, VA, CN, BAC, DA
*Limosilactobacillus fermentum*	PC-76	S, VA
*Lactobacillus johnsonii*	PI-80	CIP, S, OT, VA, CN, BAC, FUS, DA
*Lactobacillus johnsonii*	PI-83	CIP, S, OT, VA, CN, BAC, FUS, DA
*Lactobacillus johnsonii*	PI-93	CIP, S, PEN, OT, VA, CN, BAC, FUS, DA

AML: Amoxycillin, ERY: Erythromycin, CIP: Ciprofloxacin, S: Streptomycin, P: Penicillin, OT: Oxytetracycline, VA: Vancomycin, CN: Gentamicin, BAC: Bacitracin, CHL: Chloramphenicol, FUS: Fusidic acid, DA: Doxycycline.

## Data Availability

The data presented in this study are available on request from corresponding author.

## Supplementary Materials

The following supporting information can be downloaded at: https://www.mdpi.com/article/10.3390/ani13081284/s1, Table S1: MICs of different antibiotics against *Lactobacillus* isolates.

## References

[1] Ministry of Finance, Government of Pakistan (2021). Economic Survey of Pakistan.

[2] Vielitz E. (2016). Evolution of avian pathology in Europe during the past 50 years. Lohmann. Inf..

[3] Batista D.F.A., de Freitas Neto O.C., de Almeida A.M., Maboni G., de Carvalho T.F., de Carvalho T.P., Barrow P.A., Junior A.B. (2018). Evaluation of pathogenicity of *Salmonella* Gallinarum strains harbouring deletions in genes whose orthologues are conserved pseudogenes in *S.* Pullorum. PLoS ONE.

[4] Kumari D., Mishra S., Lather D. (2013). Pathomicrobial studies on *Salmonella* Gallinarum infection in broiler chickens. Vet. World.

[5] Sannat C., Patyal A., Rawat N., Ghosh R., Jolhe D., Shende R., Hirpurkar S., Shakya S. (2017). Characterization of *Salmonella* Gallinarum from an outbreak in Raigarh, Chhattisgarh. Vet. World.

[6] Revolledo L., Ferreira A.J.P. (2012). Current perspectives in avian salmonellosis: Vaccines and immune mechanisms of protection. J. Appl. Poult. Res..

[7] Kim N.H., Ko D.S., Ha E.J., Ahn S., Choi K.S., Kwon H.J. (2021). Optimized Detoxification of a Live Attenuated Vaccine Strain (SG9R) to Improve Vaccine Strategy against Fowl Typhoid. Vaccines.

[8] Rajoka M.S.R., Hayat H., Sarwar S., Mehwish H., Ahmad F., Hussain N., Shah S., Khurshid M., Siddiqu M., Shi J. (2018). Isolation and evaluation of probiotic potential of lactic acid bacteria isolated from poultry intestine. Microbiology.

[9] Joint FAO, World Health Organization (2001). Evaluation of Allergenicity of Genetically Modified Foods: Report of a Joint FAO.

[10] Pokorna A., Manakova T., Cizek A. (2019). Properties of potentially probiotic *Lactobacillus* isolates from poultry intestines. Acta. Vet. Brno.

[11] Andino A., Hanning I. (2015). *Salmonella* enterica: Survival, colonization, and virulence differences among serovars. Sci. World J..

[12] Orji M.U., Onuigbo H.C., Mbata T.I. (2005). Isolation of *Salmonella* from poultry droppings and other environmental sources in Awka, Nigeria. Int. J. Infect. Dis..

[13] Muktaruzzaman M., Haider M., Ahmed A., Alam K., Rahman M., Khatun M., Rahman M., Hossain M. (2010). Validation and refinement of *Salmonella* pullorum (SP) colored antigen for diagnosis of *Salmonella* infections in the field. Int. J. Poult. Sci..

[14] Brooks G.F., Carroll K.C., Butel J., Morse S., Mietzner T., Jawetz M. (2007). Adelberg’s medical microbiology. Sultan Qaboos Univ. Med. J..

[15] Al-Harthi M., Halawani M., Abdelkader S. (2012). Detection of *Salmonella* strains in clinical samples from Saudi Arabia by *invA* and *hilA* polymerase chain reaction (PCR)-based assays. Afri. J. Microbiol. Res..

[16] Kang M.S., Kwon Y.K., Jung B.Y., Kim A., Lee K.M., An B.K., Song E.A., Kwon J.H., Chung G.S. (2011). Differential identification of *Salmonella* enterica subsp. enterica serovar Gallinarum biovars Gallinarum and Pullorum based on polymorphic regions of *glgC* and *speC* genes. Vet. Microbiol..

[17] Asghar S., Arif M., Nawaz M., Muhammad K., Ali M., Ahmad M., Iqbal S., Anjum A., Khan M., Nazir J. (2016). Selection, characterisation and evaluation of potential probiotic *Lactobacillus* spp. isolated from poultry droppings. Benef. Microb..

[18] Nawaz M., Wang J., Zhou A., Ma C., Wu X., Moore J.E., Cherie Millar B., Xu J. (2011). Characterization and transfer of antibiotic resistance in lactic acid bacteria from fermented food products. Curr. Microbiol..

[19] McGinnis S., Madden T.L. (2004). BLAST: At the core of a powerful and diverse set of sequence analysis tools. Nucleic Acids Res..

[20] Bao Y., Zhang Y., Zhang Y., Liu Y., Wang S., Dong X., Wang Y., Zhang H. (2010). Screening of potential probiotic properties of *Lactobacillus fermentum* isolated from traditional dairy products. Food. Control..

[21] Delgado S., Osullivan E., Fitzgerald G., Mayo B. (2007). Subtractive screening for probiotic properties of *Lactobacillus* species from the human gastrointestinal tract in the search for new probiotics. J. Food. Sci..

[22] Saleem N., Nawaz M., Ghafoor A., Javeed A., Mustafa A., Yousuf M.R., Khan I. (2018). Phenotypic and Molecular Analysis of Antibiotic Resistance in Lactobacilli of Poultry Origin from Lahore, Pakistan. Pak. Vet. J..

[23] EFSA Panel on Additives and Products or Substances used in Animal Feed (FEEDAP) (2012). Guidance on the assessment of bacterial susceptibility to antimicrobials of human and veterinary importance. EFSA J..

[24] Todoriki K., Mukai T., Sato S., Toba T. (2001). Inhibition of adhesion of food-borne pathogens to Caco-2 cells by *Lactobacillus* strains. J. Appl. Microbiol..

[25] Yoon K.B., Song B.J., Shin M.Y., Lim H.C., Yoon Y.H., Jeon D.Y., Ha H., Yang S.I., Kim J.B. (2017). Antibiotic resistance patterns and serotypes of *Salmonella* spp. isolated at Jeollanam-do in Korea. Osong Public Health Res. Perspect..

[26] Sornplang P., Leelavatcharamas V., Sukon P., Yowarach S. (2011). Antibiotic resistance of lactic acid bacteria isolated from a fermented fish product, pla-chom. Res. J. Microbiol..

[27] Kerry R.G., Patra J.K., Gouda S., Park Y., Shin H.S., Das G. (2018). Benefaction of probiotics for human health: A review. J. Food Drug Analy..

[28] Aziz G., Fakhar H., ur Rahman S., Tariq M., Zaidi A. (2019). An assessment of the aggregation and probiotic characteristics of *Lactobacillus* species isolated from native (desi) chicken gut. J. Appl. Poult. Res..

[29] Mustafa A., Nawaz M., Rabbani M., Tayyab M., Khan M. (2022). Characterization and evaluation of anti-*Salmonella* Enteritidis activity of indigenous probiotic lactobacilli in mice. Open Life Sci..

[30] Khan I., Nawaz M., Anjum A.A., Ahmad M.u.D. (2019). Isolation and in vitro Characterization of Anti-*Salmonella* Enteritidis Probiotic Potential of Indigenous Lactobacilli from Poultry. Pak. Vet. J..

[31] Abbas A., Rizvi F., Hussain S., Ali S., Rafique R., Manzoor A., Waqar H., Akram R., Shaukat M., Shaukat H. (2021). Immuno-Modulatory effects of *Lactobacillus* in *Salmonella* Gallinarum Infected Broiler Chicks. Pak. J. Sci..

[32] Stephenson D.P., Moore R.J., Allison G.E. (2010). *Lactobacillus* strain ecology and persistence within broiler chickens fed different diets: Identification of persistent strains. Appl. Environ. Microbiol..

[33] Niewold T. (2015). Chapter 9: Intestinal health biomarkers in vivo. Intestinal Health: Key to Maximise Growth Performance in Livestock.

[34] Salehizadeh M., Modarressi M.H., Mousavi S.N., Ebrahimi M.T. (2020). Evaluation of lactic acid bacteria isolated from poultry feces as potential probiotic and its in vitro competitive activity against *Salmonella typhimurium*. Vet. Res. Forum.

[35] Upadhaya S., Hossiendoust A., Kim I. (2016). Probiotics in *Salmonella*-challenged Hy-Line brown layers. Poult. Sci..

[36] Bull M., Plummer S., Marchesi J., Mahenthiralingam E. (2013). The life history of *Lactobacillus acidophilus* as a probiotic: A tale of revisionary taxonomy, misidentification and commercial success. FEMS Microbiol. Lett..

[37] Khan M., Anjum A., Nawaz M., Awan A. (2020). In vitro characterization of probiotic properties and anti-*Campylobacter* activity of *Lactobacillus* spp. isolated from poultry, fermented foods and human faeces. J. Anim. Plant. Sci..

[38] Spivey M.A., Dunn-Horrocks S.L., Duong T. (2014). Epithelial cell adhesion and gastrointestinal colonization of *Lactobacillus* in poultry. Poult. Sci..

[39] Shokryazdan P., Sieo C.C., Kalavathy R., Liang J.B., Alitheen N.B., Faseleh Jahromi M., Ho Y.W. (2014). Probiotic potential of *Lactobacillus* strains with antimicrobial activity against some human pathogenic strains. Biomed. Res. Int..

[40] Arif A., Nawaz M., Rabbani M., Iqbal S., Mustafa A., Yousuf M.R., Muhammad K. (2018). Screening, characterization and physicochemical optimization of phosphorus solubilization activity of potential probiotic *Lactobacillus* spp.. Pak. Vet. J..

[41] An Y.H., Dickinson R.B., Doyle R.J. (2000). Mechanisms of bacterial adhesion and pathogenesis of implant and tissue infections. Handbook of Bacterial Adhesion.

[42] Potocnjak M., Pusic P., Frece J., Abram M., Jankovic T., Gobin I. (2017). Three new *Lactobacillus* plantarum strains in the probiotic toolbox against gut pathogen *Salmonella* enterica serotype Typhimurium. Food Tech. Biotech..

[43] Suwannaphan S. (2021). Isolation, identification and potential probiotic characterization of lactic acid bacteria from Thai traditional fermented food. AIMS Microbiol..

[44] Pessoa W.F.B., Melgaço A.C.C., de Almeida M.E., Ramos L.P., Rezende R.P., Romano C.C. (2017). In vitro activity of lactobacilli with probiotic potential isolated from cocoa fermentation against *Gardnerella vaginalis*. Biomed. Res. Int..

[45] Dowarah R., Verma A.K., Agarwal N., Singh P., Singh B.R. (2018). Selection and characterization of probiotic lactic acid bacteria and its impact on growth, nutrient digestibility, health and antioxidant status in weaned piglets. PLoS ONE.

