# Supplementary material for: In Vitro Characterization of Probiotic Potential of Limosilactobacillus fermentum against Salmonella Gallinarum Causing Fowl Typhoid
Source: Animals (Basel). 2023 Apr 8;13(8):1284. doi: 10.3390/ani13081284 (PMC10135235; doi:10.3390/ani13081284)
Supplement: Supplementary file 1 [file animals-13-01284-s001.zip › animals-2229617-supplementary.pdf]

Table.S1 MICs of different antibiotics against *Lactobacillus* isolates

Isolates	Organism	MICs (µg/mL) of different antibiotics against lactobacilli isolates											
		P	AML	VA	S	CN	ERY	CIP	CHL	OT	DA	BAC	FUS
PC-01	*Limosilactobacillus reuteri*	32	1	64	128	16	16	32	1	32	32	16	8
PC-04	*Ligilactobacillus salivarius*	1	1	64	256	16	0.25	64	8	16	32	32	1
PC-07	*Ligilactobacillus salivarius*	32	2	128	128	32	0.125	32	16	16	16	16	0.5
PC-10	*Limosilactobacillus fermentum*	4	0.5	128	64	2	0.125	32	2	2	1	1	0.5
PC-11	*Limosilactobacillus reuteri*	2	1	64	256	32	0.25	16	16	32	64	32	0.125
PC-12	*Limosilactobacillus reuteri*	2	1	128	>256	32	16	16	2	64	128	64	0.125
PC-13	*Limosilactobacillus reuteri*	16	1	128	128	64	0.25	32	1	128	128	64	1
PC-15	*Limosilactobacillus reuteri*	1	2	>256	64	32	32	32	1	64	64	32	1
PC-17	*Limosilactobacillus reuteri*	2	2	128	128	16	128	16	2	128	128	16	0.5
PC-24	*Limosilactobacillus reuteri*	1	4	128	256	16	0.5	64	16	64	32	16	0.5
PC-25	*Limosilactobacillus reuteri*	4	4	256	128	32	0.125	64	8	32	64	32	1
PC-28	*Limosilactobacillus reuteri*	16	2	128	256	32	0.25	32	1	64	32	16	1
PI-47	*Lactobacillus crispatus*	32	2	64	64	64	0.125	64	1	16	16	16	0.5
PC-55	*Lactobacillus crispatus*	16	1	128	64	128	0.25	32	2	32	32	1	0.5
PC-60	*Lactobacillus crispatus*	2	16	128	128	64	16	32	2	2	16	1	16
PC-65	*Lactobacillus crispatus*	2	2	128	128	64	0.50	16	2	32	8	16	8
PC-68	*Lactobacillus crispatus*	0.5	1	>256	64	32	32	32	1	64	16	16	0.5
PC-76	*Limosilactobacillus fermentum*	1	2	128	256	2	0.125	2	1	4	4	2	1
PI-80	*Lactobacillus johnsonii*	1	0.5	64	64	16	0.25	128	1	32	64	64	8
PI-83	*Lactobacillus johnsonii*	2	2	128	128	32	0.25	32	1	64	64	128	4
PI-93	*Lactobacillus johnsonii*	32	1	64	64	32	0.50	32	2	32	32	32	16

Breakpoints of antibiotics against *Lactobacillus* adopted are as following**; P:** Penicillin≥8, **AML:** Amoxycillin≥8, **VA:** Vancomycin≥2 or n.r, **S:** Streptomycin≥16 or 64, **CN:** Gentamicin≥8, **ERY:** Erythromycin≥1, **CIP:** Ciprofloxacin≥4, **CHL:** Chloramphenicol≥4, **OT:** Oxytetracycline≥4 or 8 or 16, **DA:** Doxycycline≥4 or 8 or 16, **BAC:** Bacitracin≥8**, FUS:** Fusidic acid>2
